# NMDA Receptor Hypofunction in the Aging-Associated Malfunction of Peripheral Tissue

**DOI:** 10.3389/fphys.2021.687121

**Published:** 2021-06-24

**Authors:** Angélica Rivera-Villaseñor, Frida Higinio-Rodríguez, Laura Nava-Gómez, Bárbara Vázquez-Prieto, Isnarhazni Calero-Vargas, Rafael Olivares-Moreno, Mónica López-Hidalgo

**Affiliations:** ^1^Escuela Nacional de Estudios Superiores, Unidad Juriquilla, Universidad Nacional Autónoma de México, Mexico City, Mexico; ^2^Facultad de Medicina, Universidad Autónoma de Querétaro, Querétaro, Mexico; ^3^Instituto de Neurobiología, Universidad Nacional Autónoma de México, Juriquilla, Mexico

**Keywords:** hypofunction of NMDA receptor, aging, skin turnover, bone remodelling, glomerulus, D-serine

## Abstract

Glutamatergic transmission through NMDA receptors (NMDARs) is important for the function of peripheral tissues. In the bone, NMDARs and its co-agonist, D-serine participate in all the phases of the remodeling. In the vasculature, NMDARs exerts a tonic vasodilation decreasing blood perfusion in the *corpus cavernosum* and the filtration rate in the renal glomerulus. NMDARs are relevant for the skin turnover regulating the proliferation and differentiation of keratinocytes and the formation of the cornified envelope (CE). The interference with NMDAR function in the skin leads to a slow turnover and repair. As occurs with the brain and cognitive functions, the manifestations of a hypofunction of NMDARs resembles those observed during aging. This raises the question if the deterioration of the glomerular vasculature, the bone remodeling and the skin turnover associated with age could be related with a hypofunction of NMDARs. Furthermore, the interference of D-serine and the effects of its supplementation on these tissues, suggest that a decrease of D-serine could account for this hypofunction pointing out D-serine as a potential therapeutic target to reduce or even prevent the detriment of the peripheral tissue associated with aging.

## NMDARs Participation in the Remodeling Process of the Bone

Bone is a connective tissue with structural and support functions providing a hard but lightweight frame to anchor the muscles. It protects the nervous system, various internal organs, and plays important roles in metabolic and homeostatic functions (Al-Suhaimi and Al-Jafary, 2020). Healthy bone tissue is constantly remodeling and requires the communication between bone-resorbing (osteoclast) and bone-forming cells (osteoblast) to maintain minerals homeostasis. This process is divided into four sequential phases: activation, resorption, reversal, and formation (Langdahl et al., 2016).

Glutamate release by chondrocytes, osteoclast and osteoblast plays an important role regulating bone remodeling through the activation of glutamate transporters, metabotropic and ionotropic receptors expressed in bone cells (Genever and Skerry, 2001; Hinoi et al., 2002; Morimoto et al., 2006). In particular, in the activation phase, NMDARs expressed in chondrocytes regulate the transduction of mechanical forces in the cartilage that can trigger the beginning of the cycle (Ramage et al., 2008). Osteocytes release paracrine factors such as Receptor Activator of Nuclear Factor κ-B Ligand (RANKL) and osteoprotegerin (OPG) to recruit and activate circulating osteoclast to prepare the bone surface with lining cells. In the resorption phase, osteoclastogenesis requires the activation of NMDAR expressed by osteoclast precursors through the induction of the nuclear translocation of NF-kappa B (Merle et al., 2003; Wu et al., 2016). Then, osteoclasts migrate to the target zone forming a sealing zone limiting the resorption of the remodeling (Nakamura et al., 1996; Wu et al., 2017). They create an acidic microenvironment to demineralize and degrade the bone matrix to remove bone products through transcytosis before entering into apoptosis (Touaitahuata et al., 2014). Before the new bone is generated, pre-osteoblasts differentiate into mature osteoblasts in the reversal phase, a process that is also dependent on NMDARs through the regulation of alkaline phosphatase activity (Dobson and Skerry, 2000). In this phase, lining cells envelop and digest collagen fibrils derivate from cavities made by osteoclast (Everts et al., 2002) to prepare the bone surface depositing collagen into the bone matrix (Abdelgawad et al., 2016). Finally, during the formation phase, osteoblasts deposit collagen type I, alkaline phosphatase, and osteocalcin in a NMDAR activity dependent manner (Hinoi et al., 2002; Brown et al., 2013). Once the cellular bone matrix is formed, osteoblast can either dedifferentiate to osteocytes (Sawa et al., 2019), convert into bone progenitors (Knopf et al., 2011), or initiated apoptosis (Jilka et al., 1998).

**FIGURE 1:**
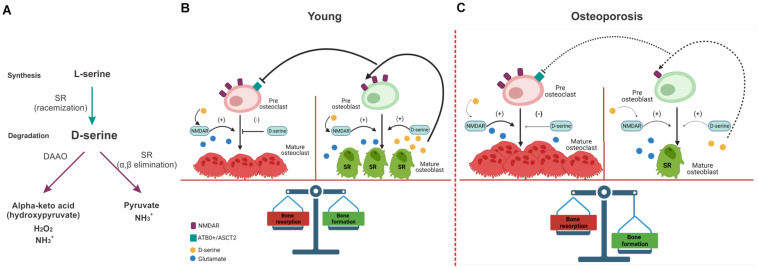
Effect of NMDAR and D-serine in bone-resorption and bone-formation processes in young animals and during osteoporosis. **(A)** Mechanisms of biosynthesis and degradation of D-serine. D-serine is synthetized from L-serine by SR. D-serine is catabolized by DAAO producing the corresponding alpha-keto acid (hydroxypyruvate), hydrogen peroxide and ammonia. D-serine can also be degradated through α,β elimination, obtaining pyruvate, and ammonia as products. **(B)** In young healthy subjects, osteoclast and osteoblast maturation is facilitated by NMDAR activity. D-serine synthetized by SR in mature osteoblast (bold lines) would supress osteoclast maturation through amino acid transportes (ARB0^+^ and ASCT2) located in pre-osteoclasts and would promote osteoblast and osteoclast maturation through the activation of NMDAR. **(C)** During the osteoporosis-associated with aging, there is a decrease in the expression of NMDAR in pre-osteoblast that would affect their maturation. This would lead to a decrease in D-serine released by osteoblast (dashed lines) and a reduction of the positive input onto the osteoblast maturation. In parallel, a reduction of D-serine would decrease the inhibition of osteoclast maturation favoring bone resorption. SR, Serine racemase; DAAO, D-amino acid oxidase.

The NR1 subunits are expressed in the bone cells and contain a site that binds glycine or D-serine that is necessary for NMDAR activation (Patton et al., 1998; Itzstein et al., 2001). In particular, D-serine is synthesized from L-serine by the action of serine racemase (SR) (Wolosker et al., 1999). Its activity is optimum at pH 8–9, being 6 times higher than in physiological pH (7.4) (Wolosker et al., 1999). The degradation of D-serine is carried out mainly by the enzyme D-amino acid oxidase (DAAO), a flavoprotein that oxidates D-serine through the reduction of the cofactor FAD that results in the corresponding alpha-keto acid (hydroxypyruvate), hydrogen peroxide and ammonia (Sacchi et al., 2013), however, it has also been reported that SR can catalyzes the degradation of D-serine through the α,β-elimination of water (Foltyn et al., 2005; Figure 1). In the bone, SR are expressed in chondrocytes (Takarada et al., 2009) and osteoblast, however, no expression of DAAO was found in culture osteoblast and osteoclast (Takarada et al., 2012) suggesting that D-serine catabolism could occur in the kidney. Besides D-serine effects regulating NMDAR activity, it can act through amino acid transporters, ATB0^+^ and ASCT2, to promote the differentiation and maturation of osteoclasts. Because D-serine is released by osteoblast and does not change osteoblastogenesis, a paracrine effect of osteoblast-derived D-serine onto neighboring osteoclast has been proposed (Takarada et al., 2012) suggesting that in physiological conditions, D-serine could be inhibiting the bone-resorbing process (Figure 1).

In physiological conditions, NMDARs activation and D-serine are necessary to maintain a balance between bone-formation and absorption processes, and could account for alterations as occurs with aging. Aged bone is characterized by an unbalance toward bone-resorbing process that leads to a progressive decline in the bone mineral density (BMD) leading to osteoporosis. During aging, there is a trend to reduce physical activity that contributes to the decrease of a bone-formation process (Langdahl et al., 2016). In a rat model of disuse osteoporosis induced by mechanical unloading, Ho et al. (2005) reported a decrease of BMD, trabecular volume and osteogenic gene expression (collagen type 1 and cbfa1/Runx2). These changes were accompanied with a decrease in NR1 and NR2 subunits of NMDARs in osteoblasts but not in osteoclasts. Furthermore, an antagonist of NMDARs mimicked the down-regulation of osteogenic genes observed in disused bones. This evidence suggests that in this osteoporosis, NMDAR activity could be responsible for the decrease in BMD due an interference with osteoblast function. Furthermore, it suggests that mechanical stress due to physical activity is necessary for NMDAR expression in osteoblast.

In parallel, the decrease in BMD induced in an ovariectomized mice (a model of postmenopausal osteoporosis), glutamate administration (i.p.) reduced the BMD lost in compare with ovariectomized control mice (Hinoi et al., 2007). The authors proposed that glutamate would promote osteoblastogenesis mediated by Runx2 through NMDARs activation and may suppress osteoclastogenesis mediated by NF-kB through the cystine/Glu antiporter located in osteoclasts (Yoneda, 2017). This result shows the relevance of NMDARs activity in osteoporosis and suggest that in aged subjects, the decrease in BMD induced by a decrease in physical activity or sexual hormones in females would lead to a hypofunction of NMDARs in osteoblast and hence a reduction in mature osteoblast. This would reduce the synthesis of D-serine by osteoblast and would reduce the inhibition of D-serine on osteoclastogenesis favoring resorption process and hence osteoporosis (Figure 1) (Steinert and Marekov, 1999; Candi et al., 2001; Kalinin et al., 2002).

## NMDARs as a Regulator of Peripheral Vasculature

The vascular system is made up of vessels whose main function is to supply nutrients, oxygen, and to remove waste products (Tennant and McGeachie, 1990; Eble and Niland, 2009). The vascular endothelium is a dynamic element that responds to vasoconstrictive and vasodilatory factors (Duffy et al., 1998) like nitric oxide (NO) (Michel and Vanhoutte., 2010; Gradinaru et al., 2015). In particular, NO is synthetized by the endothelial nitric oxide synthase (eNOS) and depends on the association of Ca^2+^-calmodulin to activate the eNOS and convert L-arginine in NO. This gas diffuses across the endothelium to the smooth muscle where it increases the levels of cyclic guanosine monophosphate (cGMP) and reduces the smooth muscle tension causing vasodilation (Sandoo et al., 2010). In the central nervous system, a major regulator of eNOS activity are NMDARs (LeMaistre et al., 2012; Lu et al., 2019). In the brain endothelial cells, NMDAR activation induces vasodilation through the increase in NO production in a D-serine concentration-dependent manner (LeMaistre et al., 2012; Lu et al., 2019).

In addition to the above-mentioned functions of the vasculature, there are arrangements of vessels that account for specific functions, for example, in the glomerulus of the kidney, blood vessels determine the filtration rate of the blood to produce the urine. Here, glutamate exocytosis from mature podocytes can modulate in an autocrine/paracrine manner the activity of NMDAR located in the podocytes (Giardino et al., 2009) to regulate vascular tone. This was first revealed by Deng and colleagues, in 2002 where they showed the expression of NR1 subunit of NMDAR (Deng et al., 2002). The blockade of this site with DCKA or blocking the NMDARs with MK-801, cause a significant vasoconstriction, a decrease in the renal blood flow and a reduction of the glomerular filtrate rate (Blantz et al., 2002). This suggests that tonic NMDAR activation vasodilates glomerular capillaries increasing renal blood flow (Deng and Thomson, 2009). Although SR has been reported in the kidney convoluted tubules (Xia et al., 2004), the glomeruli does not expressed SR (Xia et al., 2004) suggesting that plasmatic D-serine could act on glomerular NR1 subunit. DAAO mRNA and the protein is abundant in proximal tubules of the kidney (Koibuchi et al., 1995; Sasabe et al., 2014), however, no enzyme activity was found in glomeruli (Chan et al., 1979). This suggest that NMDAR in the glomeruli would be under the regulation of the levels of D-serine in the blood.

In the glomerulus, the vascular side is covered by endothelial cells separated from the urinary space by the basement membrane that is enfolded by podocytes (Wiggins, 2012) and whose functions in the filtration barrier depend on NMDAR activity. Here, the interference of podocytes glutamate exocytosis in Rab3A/KO mice, induced a disorganization in the structure of podocytes foot process and macroalbuminuria. Furthermore, MK-801 increases albumin permeability, and remodel podocyte cytoskeleton decreasing actin and myosin/IIA as well as nephrin. These structural changes were accompanied with proteinuria (Giardino et al., 2009). In immortalized podocytes, NMDA-mediated currents were strongly potentiated by D-serine, but not by glycine suggesting that D-serine is the endogenous co-agonist of NMDAR in the glomerulus (Anderson et al., 2011).

The effect of NMDAR on the physiology of the kidney is not restricted to the glomerulus, it also modulates proximal tubular reabsorption (Slomowitz et al., 2004; Deng and Thomson, 2009) and is involved in the reno-renal reflex. In the renal pelvi, NR1 subunit expressed in afferent nerves, acts as a mechanoreceptor detecting increases in intrapelvic pressure. NMDAR activation increases afferent renal nerve activity and substance P release that induces diuresis and natriuresis. Moreover, intra-pelvic administration of D-serine mimicked NMDAR activation inducing renal sensory activation. In the renal pelvis SR is expressed in the muscle and in the uroepithelial layer suggesting a local effect of D-serine in physiological conditions (Ma et al., 2008).

In the *corpus cavernosum* of the penis, the vascular tone is essential for erection. In a flaccid state, the smooth muscles are tonically contracted allowing a small amount of arterial flow, an increase in the blood flow would produce erection. Here, penil neuronal NOS (PnNOS), NR1 and NR2 subunit of NMDAR are expressed in the cavernosa nerves where they are often colocalized (Magee et al., 2003). NMDARs activation induce a non-adrenergic non-cholinergic neurogenic relaxation of the *corpus cavernosum* reinforcing a tonic vasodilator effect of NMDAR on the vasculature (Gonzalez-Cadavid and Raifer, 2000; Ghasemi et al., 2010).

DAAO and SR are expressed in the *corpus cavernosum* and cavernosal membrane, respectively (Ghasemi et al., 2010; Kim et al., 2019) suggesting local regulation of NMDAR activity. In fact, D-serine administration induces a dose-dependent and NMDAR dependent relaxation of cavernosal tissue pre-contracted with phenylephrine. This effect was blocked by an Inhibitor of NOS (Ghasemi et al., 2010; Montesinos and Mani, 2016), although an inhibition of SR by NO has been shown in cultures of neurons (Watanabe et al., 2016), these results reinforce the interaction of NO and NMDAR in vasodilation. Further experiments are required to analyze the role of NMDAR and D-serine in the erection in awake animals and points out possible therapeutic targets in the treatment of impotence.

Renal damage during aging is associated with podocytes dysfunction that can lead to matrix accumulation and glomeruloesclerosis that is manifested with a decrease in the blood flow, filtration rate, and an increase in the permeability causing proteinuria (Wiggins, 2012; Denic et al., 2016). All of this process depends on the proper function of NMDAR (Deng and Thomson, 2009; Giardino et al., 2009). In agreement with this, in mice kidneys homogenates there is an increase of calmodulin during aging, that acts as an inhibitor of NR1 subunit of NMDAR.

Aging is also associated with erectile dysfunction, a condition related with hypertension, decreased levels of testosterone, cardiovascular disease among many others. It has also been proposed that can be caused by a decrease in the effect of NO which is supported by a decrease in the NOS-containing nerve fibers in the penile of aged rats with erectile dysfunction (Gonzalez-Cadavid and Raifer, 2000). In this sense, Magee et al. (2003) did not find changes in the mRNA levels of PnNOS, nNOS and NMDAR in the penis of aged rats. However, further experiments are required to analyze the effect of aging on the levels of the proteins and the functionality of the NOS and NMDAR to rule out the involvement of NMDAR/NO expressed in cavernous nerves in erectile dysfunction. This is possible because cavernous nerves are required for the erectile responses mediated by hypothalamic medial preoptic area (MPOA) stimulation in adult male rats (Giuliano et al., 1997).

## Role of NMDARs in Skin Turnover

**FIGURE 2:**
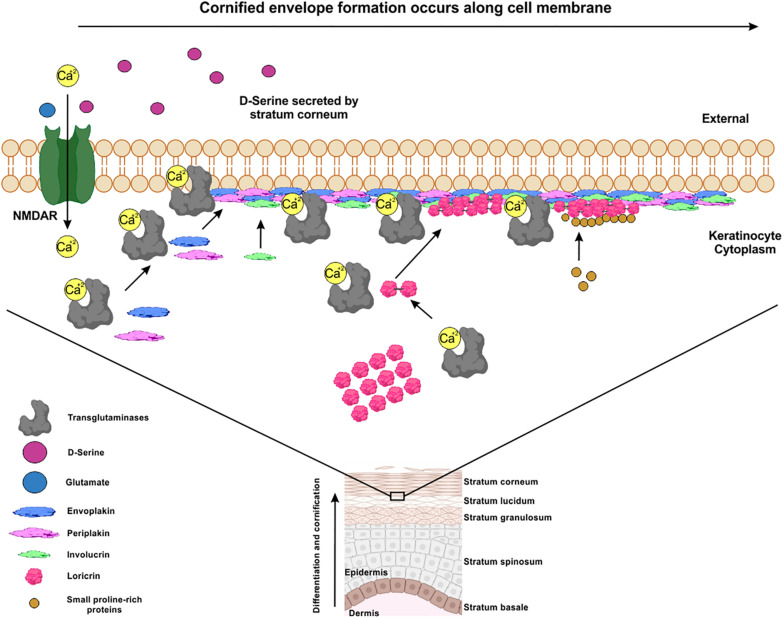
Early stages of the cornified envelope formation are calcium-deppendent. The crosslinking between Envoplakin, Periplakin, Involucrin, Loricrin and Small-prolin-rich proteins, are mediated by calcium-dependent TGs through the formation of covalent bonds. This resulted in the formation of a layer along the entire inner surface of the plasma membrane, forming a scaffold to which other precursors are added to form a mature cornified envelope (CE). D-serine secreted in stratum corneum by corneocytes can activate NMDAR expressed in keratinocytes in a paracrine way improving the differentiation and cornification of skin.

Skin is the first physical and anatomic barrier providing protection to the organism against environmental factors and agents such as UV radiation, heat, water loss, pathogens, etc. It is composed of three layers, the epidermis, the dermis, and the hypodermis. The epidermis is a self-renewing stratified and cornified epithelium divided into four layers: basal, spinous, granular, and the stratum corneum (Eckert et al., 2005). In the epidermis, keratinocytes are formed in the basal layer through cell division to replace terminally differentiated keratinocytes named corneocytes. The differentiation of the keratinocytes depends on the transglutaminases enzymes that catalyze covalent cross-linking of constituting proteins such as involucrin (IVL), loricrin (LRC), envoplakin (EVPL), periplakin (PPL), and small proline-rich proteins (SPRs) (Figure 2; Candi et al., 2005; Streubel et al., 2017). This calcium mediated crosslinking process contributes to the formation of the early cornified envelope (CE), that it is required to retain water and to limit the entry of the microbes and most chemicals through the skin (Kolarsick et al., 2011; Gilaberte et al., 2016).

NMDAR subunits are expressed in the *stratum basale*, *spinosum*, and *granulosum* of the epidermis (Fischer et al., 2004). Epidermal keratinocytes in the granular and cornified layers expressed SR localization (Inoue et al., 2014), suggesting an autocrine/paracrine effect of D-serine on the NMDAR expressed in the keratinocytes. Despite the detection of NMDAR subunits and SR in epidermal tissue, no activity of DAAO was detected (Jensen and Jacobsen, 1984). NMDRAs are involved in keratinocytes differentiation, proliferation (Fischer et al., 2004) and to maintain skin barrier and repair processes (Fuziwara et al., 2003). Cultured human keratinocytes treated with MK-801, showed a decrease in the proliferation and an increase in apoptosis (Fuziwara et al., 2003; Morhenn et al., 2004). In the same way, mice lacking serine racemase (SR-KO mice), showed significantly higher expression of filaggrin, involucrin, loricrin, and transglutaminase 3 in keratinocytes compared to control mice (Inoue et al., 2014). This suggests that a hypofunction of NMDAR would decrease the activity of the calcium dependent TGs leading to the accumulation of proteins necessary for the formation of the CE (Figure 2). In fact, in an assay of a barrier recovery, SR-KO mice had lower recovery rates after tape stripping than wild type mice revealing an important role of D-serine in the healing process of the skin.

Skin aging is a complex biological process influenced by a combination of intrinsic and extrinsic factors (Cevenini et al., 2008). These factors lead together to structural and physiological alterations in the skin (Uitto, 1997; Shin et al., 2005). In particular, there is a decrease in the capacity of keratinocytes to proliferate (Yaar and Gilchrest, 2001), the epidermis thins and the rate of the turnover of the skin slows dramatically (Suter-Widmer and Elsner, 1996; Velarde et al., 2012). This contributes to a slow healing of minor injuries and weaker surgical scars. In this sense, tape stripping studies have revealed decreased cohesiveness in aged skin (Elias et al., 2002). In fact, barrier perturbation occurred after 18 ± 2 stripping in aged skin vs. 31 ± 5 stripping in control skin (Ghadially et al., 1995).

The alterations in the epidermis during aging (Yaar and Gilchrest, 1999) and the changes observed by interfering with NMDAR are very similar, suggesting a possible relationship between aging and a hypofunction for NMDAR. Furthermore, because SR and D-serine are required for the differentiation and maintenance of the physiological function of the skin (Inoue et al., 2014), it would be interesting to analyze the potential therapeutic effect of D-serine to overcome the changes in keratinocytes differentiation caused by aging.

## Role of D-Serine in the Hypofunction of NMDARs in the Periphery

Aging is a natural process that leads to reductions in maximal function and reserve capacity in all organ systems. Changes in the function of peripheral tissue due to aging result in an increase of susceptibility to and frequency of disease, frailty, or disability. In fact, advancing age is the major risk factor for several chronic diseases in humans. Here, we provide evidence that the hypofunction of NMDARs resembles many manifestations observed during aging such as disturbed glomerular filtration, alterations in the skin turnover and bone remodeling, suggesting that NMDARs hypofunction could be associated with deterioration due to aging. We propose that a decrease in D-serine could account for this hypofunction because (1) NMDAR hypofunction in the brain associated with aging is related with a decrease in the synthesis of D-serine and not glycine (Mothet et al., 2006; Billard, 2018); (2) There is an increase of DAAO in the plasma associated with age (Lin et al., 2017); (3) NMDAR expressed in glomerular podocytes are insensitive to glycine (Anderson et al., 2011); (4) NMDAR activation induces vasodilation through the increase in NO production in a D-serine concentration-dependent manner (LeMaistre et al., 2012; Lu et al., 2019); (5) D-serine administration induces a dose-dependent and NMDAR dependent relaxation of cavernosal tissue pre-contracted with phenylephrine (Ghasemi et al., 2010); and (6) SR and NMDAR are localized in the keratinocytes of the skin (Inoue et al., 2014).

There are controversies regarding changes in plasma levels of D-serine associated with age that could account for a generalized hypofunction of NMDAR in the periphery. While some observed a negative association between D-serine levels and age (Avellar et al., 2016) others have observed an increase (Lin et al., 2017). However, plasma D-serine levels correlates with CSF or brain levels associated with malfunctioning of NMDAR in different pathologies like Schizophrenia and ALS (Hashimoto et al., 2003), and this does not occur with glycine plasma levels (Ohnuma et al., 2008; Lin et al., 2017). Although further experiments are required to analyze a decrease of D-serine in the blood with age, this evidence suggests that a decrease in D-serine levels in the brain could be reflected in blood levels. Furthermore, the quantification of local D-serine, SR and DAAO expression associated with aging in different tissues would shed light on the role of D-serine as the element regulating hypofunction on NMDAR in the periphery.

NMDAR containing NR1 subunit require both, the agonist and co-agonist, for the activation (Paoletti et al., 2013). This would limit an overactivation of NMDAR followed by D-serine supplementation in one side, however, in the other side, if glutamate is also decrease in the periphery during aging it may not be D-serine sufficient to restore NMDAR hypofunction. D-Serine supplementation can cause necrosis of proximal straight tubules when administered at high doses (800 mg/kg) (Ganote et al., 1974), but it is well tolerated at low doses (30 mg/kg). This is important to take in consideration regarding a possible therapeutic supplementation of D-serine, in this sense it will be interesting to analyze the effect of low-doses in middle aged as a strategy to prevent a detriment with less risk of possible toxicity in the kidney.

D-serine can also be absorbed from the diet, especially from milk products (Brückner et al., 1992; Csapo et al., 2009) and synthesized by microorganisms of the intestinal microbiota, such as *Firmicutes, Clostridia, Clostridiales, Lachnospiraceae*, and *Eisenbergiella* (Matsumoto et al., 2018; Nakade et al., 2018). Supplementation with prebiotics such as fructo-oligosaccharides and galacto-oligosaccharides increased serum levels of D-serine and the expression of the NR1 and NR2A subunits in the prefrontal cortex (Savignac et al., 2013). These findings raise the possibility of a restauration of D-serine levels with the diet to reduced NMDAR hypofunction. However, is not clear how intestinal D-serine transporters changes with aging, and would be important to determine the physiological and pathological levels of plasmatic D-serine.

## Data Availability Statement

The original contributions presented in the study are included in the article/supplementary material, further inquiries can be directed to the corresponding author/s.

## Author Contributions

ARV, ROM, and MLH edited the article. All authors drafted the article and approved the final version.

## Conflict of Interest

The authors declare that the research was conducted in the absence of any commercial or financial relationships that could be construed as a potential conflict of interest.
